# Mother–infant interaction in women with depression in pregnancy and in women with a history of depression: the Psychiatry Research and Motherhood – Depression (PRAM-D) study

**DOI:** 10.1192/bjo.2021.52

**Published:** 2021-05-25

**Authors:** Rebecca H. Bind, Alessandra Biaggi, Aoife Bairead, Andrea Du Preez, Katie Hazelgrove, Freddie Waites, Susan Conroy, Paola Dazzan, Sarah Osborne, Susan Pawlby, Vaheshta Sethna, Carmine M. Pariante

**Affiliations:** Department of Psychological Medicine, Institute of Psychiatry, Psychology and Neuroscience, King's College London, UK; Department of Psychological Medicine, Institute of Psychiatry, Psychology and Neuroscience, King's College London, UK; Minds In Mind, Ireland; Department of Psychological Medicine, Institute of Psychiatry, Psychology and Neuroscience, King's College London, UK; and The Maurice Wohl Clinical Neuroscience Institute, Department of Basic and Clinical Neuroscience, Institute of Psychiatry, Psychology and Neuroscience, King's College London, UK; Department of Psychological Medicine, Institute of Psychiatry, Psychology and Neuroscience, King's College London, UK; Department of Psychological Medicine, Institute of Psychiatry, Psychology and Neuroscience, King's College London, UK; Department of Psychological Medicine, Institute of Psychiatry, Psychology and Neuroscience, King's College London, UK; Department of Psychological Medicine, Institute of Psychiatry, Psychology and Neuroscience, King's College London, UK; Department of Psychological Medicine, Institute of Psychiatry, Psychology and Neuroscience, King's College London, UK; Department of Psychological Medicine, Institute of Psychiatry, Psychology and Neuroscience, King's College London, UK; Sackler Institute for Translational Neurodevelopment, Department of Forensic & Neurodevelopmental Sciences, Institute of Psychiatry, Psychology and Neuroscience, King's College London, UK; Department of Psychological Medicine, Institute of Psychiatry, Psychology and Neuroscience, King's College London, UK

**Keywords:** Perinatal psychiatry, depressive disorders, developmental disorders, psychosocial interventions, childhood experience

## Abstract

**Background:**

Little is known about the effects of depression before birth on the quality of the mother–infant interaction.

**Aims:**

To understand whether depression, either in pregnancy or in lifetime before pregnancy, disrupts postnatal mother–infant interactions.

**Method:**

We recruited 131 pregnant women (51 healthy, 52 with major depressive disorder (MDD) in pregnancy, 28 with a history of MDD but healthy pregnancy), at 25 weeks’ gestation. MDD was confirmed with the Structured Clinical Interview for DSM-IV Disorders. Neonatal behaviour was assessed at 6 days with the Neonatal Behavioural Assessment Scale, and mother–infant interaction was assessed at 8 weeks and 12 months with the Crittenden CARE-Index.

**Results:**

At 8 weeks and 12 months, dyads in the depression and history-only groups displayed a reduced quality of interaction compared with healthy dyads. Specifically, at 8 weeks, 62% in the depression group and 56% in the history-only group scored in the lowest category of dyadic synchrony (suggesting therapeutic interventions are needed), compared with 37% in the healthy group (*P* = 0.041); 48% and 32%, respectively, scored the same at 12 months, compared with 14% in the healthy group (*P* = 0.003). At 6 days, neonates in the depression and history-only groups exhibited decreased social-interactive behaviour, which, together with maternal socioeconomic difficulties, was also predictive of interaction quality, whereas postnatal depression was not.

**Conclusions:**

Both antenatal depression and a lifetime history of depression are associated with a decreased quality of mother–infant interaction, irrespective of postnatal depression. Clinicians should be aware of this, as pregnancy provides an opportunity for identification and intervention to support the developing relationship.

Depression throughout the perinatal period is critical in determining risk to offspring mental health outcomes,^1^ and a disrupted mother–infant interaction is a possible mechanism underpinning this transmission.^2^ Most research has focused on postnatal depression (PND), which can manifest as unresponsiveness to, and withdrawal from the infant, or as intrusive and controlling behaviour,^3,4^ with concomitant reduced sensitivity.^2,5^ Less well-studied, however, is whether depression during the antenatal period – affecting up to 20% of pregnancies^6^ – can also interfere with the quality of the mother–infant interaction. Indeed, studies have shown that the association between PND and a less optimal mother–infant interaction may be attributable to a continuation of impaired foetal attachment from pregnancy into the postnatal period,^7^ and that antenatal depressive symptoms are associated with poorer bonding and increased maternal unresponsiveness postnatally.^8–10^ However, these studies have used self-report measures of mood symptoms, largely in community samples. To our knowledge, no prior studies have looked at mother–infant interactions in the context of clinically significant major depressive disorder (MDD), confirmed through a structured, standardised diagnostic interview, in pregnant women recruited from perinatal mental health services. It is thus important to fill this gap, as antenatal depression at a clinical level may have a more severe effect on the dyadic interaction.

Of note, there is also little evidence on whether a lifetime history of MDD alone can affect mother–infant interactions, in the absence of depression in pregnancy. Only one study, to our knowledge, has examined this, and found that mothers (and fathers) with a history of depression were more likely to display negative affect when interacting with their infants, even if they were well in the perinatal period.^11^ Given that a history of MDD is one of the largest risk factors for perinatal MDD,^6^ and previous literature finds associations between perinatal MDD and difficulties in the interaction, it is worth investigating whether these difficulties may be present even in women with a past history of depression who do not necessarily meet clinical criteria for a depressive episode during pregnancy, either because of subsyndromal symptom carryover into pregnancy, or because a past history may interfere with biological mechanisms underpinning the mother–infant bond.^12,13^

Finally, it is important to establish whether any putative effects of maternal depression on the mother–infant interaction are confounded by other maternal and infant variables that are relevant to the mother–infant interaction, such as maternal childhood maltreatment and socioeconomic stress, which not only predispose women to later depression,^14,15^ but are also associated with decreased maternal sensitivity;^16,17^ or PND, which is associated with both depression in pregnancy and a history of depression,^6^ and is thought to affect maternal sensitivity;^5^ or suboptimal neonatal behaviour, which has been associated with both antenatal depression^18^ and decreased dyadic synchrony.^19^ Of note, although prior studies have assessed the effect of maternal depression on separate maternal and infant domains of behaviour, the impact on dyadic synchrony – a precursor to infant attachment that reflects behavioural patterns of both members of the dyad together as one unit – has not been previously studied.

## Current study

Thus, the novelty of our objectives are as follows: we examined maternal depression before birth (either during pregnancy or lifetime before pregnancy); we studied mother–infant interaction, both through dyadic synchrony and through separate maternal and infant domains of behaviour; we assessed the interaction at two time points (8 weeks and 12 months) in the first postnatal year; and we tested whether women with depression presented with clinical or sociodemographic risk factors that may have contributed to the putative association between maternal depression and difficulties in the mother–infant interaction.

We overall hypothesized that (a) MDD experienced during or before pregnancy would be associated with decreased dyadic synchrony across the postnatal period and (b) based on previous literature above, possible confounders of this relationship may be maternal history of childhood maltreatment, maternal socioeconomic difficulties, maternal PND and suboptimal infant neonatal behaviour.

## Method

### Design

The present study is part of the Psychiatry Research and Motherhood – Depression (PRAM-D) study,^18^ a prospective longitudinal study of women in pregnancy and the postpartum, and their offspring. The sample included 131 women: 51 healthy women, attending their routine antenatal ultrasound scan at King's College Hospital [healthy group]; 52 women diagnosed with depression, referred to the Maudsley Perinatal Psychiatry Services [depression group]; and 28 ‘history-only’ women, who had a history of MDD but no current diagnosis, recruited from either their regular antenatal scan or the psychiatric clinical service (where they were referred for assessment only, because of the historical vulnerability) [history-only group]. Women and their offspring were assessed from 25 weeks’ gestation (baseline) until 1 year postnatal.

The authors assert that all procedures contributing to this work comply with the ethical standards of the relevant national and institutional committees on human experimentation and with the Helsinki Declaration of 1975, as revised in 2008. All procedures involving human patients were approved by the King's College Hospital Research Ethics Committee (approval number REC 07/Q0703/48). All participants provided written informed consent.

### Participants and procedure

Inclusion criteria for the study were as follows: women of at least 18 years with a singleton pregnancy; for the depression group, a DSM-IV diagnosis of MDD in the current pregnancy, at or before 25 weeks’ gestation, as maternal responsiveness is thought to solidify by then;^20^ for the history-only group, a history of MDD but no diagnosis of MDD throughout the entire pregnancy (if women developed depression in pregnancy after 25 weeks', they were excluded from analyses); and, for healthy women, no current or past DSM-IV diagnoses. Exclusion criteria were as follows: uterine anomaly, obstetric complications, severe or relevant chronic medical conditions, history of psychosis or bipolar affective disorder and antidepressant usage at baseline (but not before or after baseline).

**Fig. 1:**
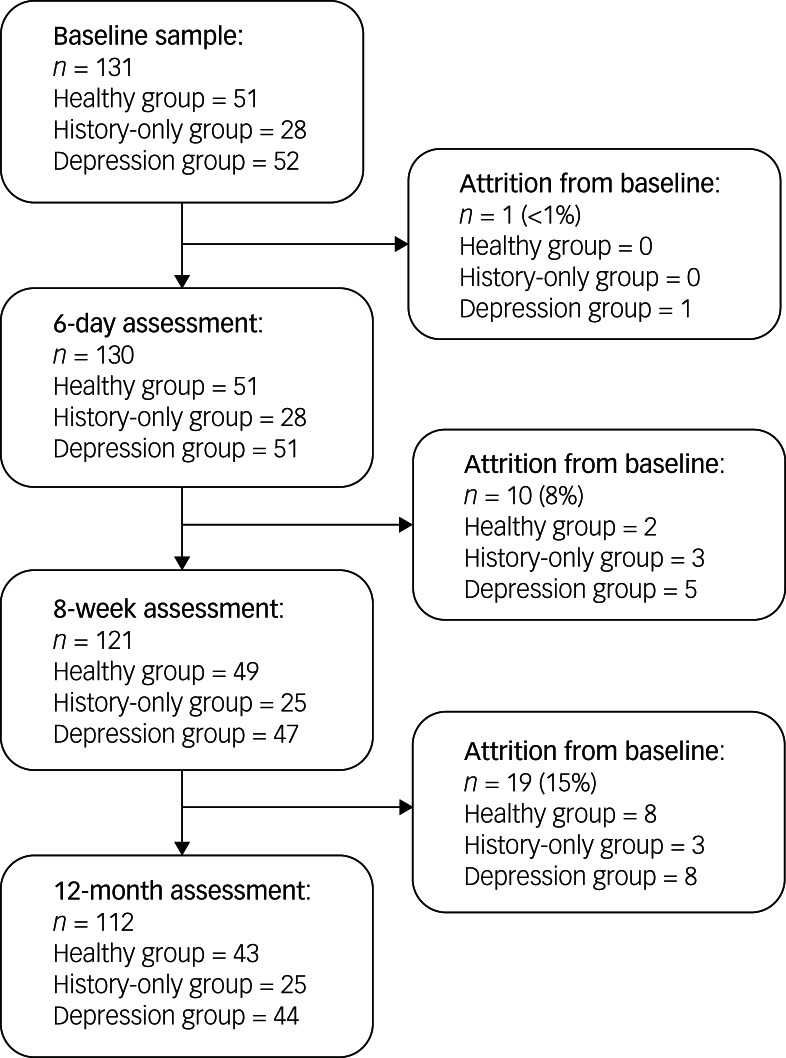
Psychiatry Research and Motherhood – Depression (PRAM-D) study participant flow chart.

Of the 131 women assessed at baseline for sociodemographic and clinical information, 130 dyads were seen at 6 days postnatal to assess neonatal behaviour, 121 dyads were seen at 8 weeks postnatal and 112 dyads were seen at 12 months postnatal, to assess sociodemographic and clinical variables, as well as the mother–infant interaction (see study flow chart in Fig. 1). There were no significant differences in attrition rates between the three groups (see Supplementary Table 1 available at https://doi.org/10.1192/bjo.2021.52), nor in socioeconomic variables or clinical variables between participants who remained in the study and those who dropped out (see Supplementary Table 2).

### Sociodemographic and clinical measures

Sociodemographic and socioeconomic (SES) variables were ascertained with a semi-structured interview. As SES variables were very highly correlated with each other, a composite SES score was created to explore the possibility of cumulative risk, as has been previously done.^21^ It encompassed maternal age, ethnicity (white versus ethnic minority), marital status (married or cohabiting versus single with or without partner), occupation (employed or student versus non-employed or full-time mother) and qualification level (GCSE or lower versus A-level or higher), all assessed at baseline, whereby a score of 0 represented the mean status across the sample, a negative score represented more risk factors for adversity and a positive score represented more protective factors. These particular SES variables were chosen as young age, belonging to an ethnic minority group, being single, being unemployed and having lower education qualifications have all been previously associated with maternal depression.^6^

#### Maternal history of childhood maltreatment

Measures of maternal history of sexual, physical and emotional abuse, and neglect were obtained with the cut-off B of the Childhood Experience of Care and Abuse Questionnaire (CECA-Q),^22^ which has been used extensively in this and other cohorts.^23^ Outcomes were based on experiences that occurred in childhood from age 0 to 17 years. Presence of childhood maltreatment was rated based on severity and frequency, according to guidelines published by Bifulco et al.^22^ Specifically, the CECA-Q contains two different cut-offs (A and B) for each of the different types of maltreatment, derived from validation against the interview measure.^22^ Cut-off B has been suggested as the optimal one to use for adulthood disorders, and thus was appropriate for our study. Cut-off points for cut-off B of each maltreatment type are described below.

##### Physical abuse

Participants who responded yes to the screening question were asked follow-up questions, including how often this abuse occurred and if they were injured as a result. Experience of physical abuse was rated separately for each parent figure, and a score was generated for each parental figure, ranging from 0 to 4. Scores of 0–2 were then recoded as 0 (minimal physical abuse); scores of 3 or 4 were recoded as 1 (severe physical abuse).

##### Sexual abuse

If participants answered yes, a total score for the first unwanted sexual experience was created by summing responses, and each unwanted sexual experience was rated 0–5; furthermore, scores of 0 or 1 were recoded as 0 (minimal sexual abuse) and scores of 2–5 were recoded as 1 (severe sexual abuse).

##### Antipathy and neglect

Antipathy and neglect were assessed with 16 self-report items about either parent in childhood. Each item was rated on a five-point Likert scale from 1 (yes definitely), to 5 (no, not at all). Eight items assessed antipathy and the other eight items measured neglect, with scores ranging from 8 to 40. For antipathy, scores of 8–27 were recoded as 0, no antipathy; scores of ≥28 were recoded as 1, severe antipathy. For parental neglect, scores of 8–24 were recoded as 0, no neglect; scores of ≥25 were recoded as 1, severe neglect.

#### Diagnosis of depression

Past and current Axis I diagnoses were assessed at baseline, using the Structured Clinical Interview for DSM-IV Disorders (SCID-I),^24^ for which a diagnosis of MDD was given if the clinical criteria for depression were met. Participants were readministered the SCID-I at 8 weeks and 12 months postnatal, to assess for episodes of PND since the previous assessment. Additionally, self-report questionnaires (Beck Depression Inventory (BDI)^25^ and State-Trait Anxiety Inventory (STAI)^26^) were collected to assess current depressive and anxious symptoms.

#### Neonatal behaviour

Neonatal behaviour was evaluated at 6 days postnatal, using the Neonatal Behavioural Assessment Scale (NBAS).^27^ The NBAS assesses neurobehavioural function in neonates up to 2 months of age, and produces information based on 28 behavioural items and 18 reflex items. These items are then divided into five clusters of functioning, covering key developmental areas, and include regulation of the autonomic system, motor system, regulation of state, range of state and orientation (the infant's ability to attend to visual and auditory stimuli, as well as their overall quality of alertness; also referred to as social-interactive abilities). For the purposes of this study, we only include the orientation cluster of the NBAS, as it assesses an infant's quality of alertness, is indicative of social-interactive abilities with the examiner and has previously been associated with dyadic interaction.^28^ Data for the healthy women and women with depression in an overlapping sample have been published before,^18^ but we present new data from the history-only group (see Results and Table 1) and its association with mother–infant interaction

**Table 1:** Sample characteristics

**Sample characteristics at baseline**					
	**Healthy group (*n* = 45–51)**	**History-only group (*n* = 27–28)**	**Depression group (*n* = 47–52)**	**Statistical test and significance**	***Post hoc* differences**
Age, years, mean (s.d.)	31.9 (4.4)	32.8 (5.9)	31.0 (6.6)	*F*(2, 128) = 0.95, *P* = 0.39	–
Ethnicity, white, *n* (%)	40 (78.4)	25 (89.3)	33 (63.5)	***χ*^2^(2) = 7.02, *P* = 0.030**	History-only *v.* depression
Education, A level or higher, *n* (%)	47 (92.2)	22 (78.6)	35 (67.3)	***χ*^2^(2) = 9.73, *P* = 0.008**	Healthy *v.* depression
Employment status, employed, *n* (%)	45 (88.2)	23 (82.1)	32 (61.5)	***χ*^2^(2) = 10.82, *P* = 0.04**	Healthy *v.* depression
Marital status, married or cohabiting, *n* (%)	45 (88.2)	23 (82.1)	28 (53.8)	***χ*^2^(2) = 18.27, *P* = 0.001**	Healthy *v.* depression, history-only *v.* depression
SES score, mean (s.d.)	0.45 (0.7)	0.24 (0.7)	−0.33 (1.2)	***H*_(2)_ = 11.08, *P* = 0.004**	Healthy *v.* depression
Number of past episodes of MDD, >2, *n* (%)	–	17 (63.0)	45 (57.7)	*χ*^2^(1) = 4.08, *P* = 0.13	–
Antidepressant usage in pregnancy, yes, *n* (%)	–	6 (21.4)	19 (36.5)	*χ*^2^(1) = 1.93, *P* = 0.16	–
Parity, primiparous, *n* (%)	26 (51.0)	17 (60.7)	23 (44.2)	*χ*^2^(2) = 6.68, *P* = 0.15	–
BDI score at baseline, mean (s.d.)	3.6 (2.5)	5.2 (3.8)	19.8 (12.9)	***F*(2, 120) = 51.44, *P* < 0.001**	Healthy *v.* depression, history-only *v.* depression
STAI-S score at baseline, mean (s.d.)	26.9 (6.8)	34.2 (11.1)	50.0 (13.1)	***F*(2, 120) = 59.10, *P* < 0.001**	Healthy *v.* history-only, healthy *v.* depression, history-only *v.* depressed
Maternal history of childhood maltreatment, yes, *n* (%)	7 (15.6)	14 (51.9)	30 (62.5)	***χ*^2^(2) = 22.19, *P* < 0.001**	Healthy *v.* history-only, healthy *v.* depression
**Sample characteristics in the postnatal period**					
	**Healthy group (*n* = 49–51)**	**History-only group (*n* = 24–28)**	**Depression group (*n* = 47–51)**	**Statistical test and significance**	***Post hoc* differences**
Mode of delivery, vaginal, *n* (%)	37 (75.5)	17 (70.8)	36 (78.3)	*χ*^2^(2) = 0.63, *P* = 0.96	–
Infant sex, female, *n* (%)	23 (46.9)	10 (41.7)	22 (46.8)	*χ*^2^(2) = 0.21, *P* = 0.90	–
NBAS orientation cluster at 6 days, mean (s.d.)	7.68 (0.9)	6.41 (1.7)	6.30 (1.6)	***F*(2, 127) = 14.60, *P* < 0.001**	Healthy *v.* history-only, healthy *v.* depression
Infant age in days at 8-week assessment, mean (s.d.)	68.69 (15.0)	81.30 (24.9)	79.00 (24.2)	***H*_(2)_ = 7.26, *P* = 0.027**	Healthy *v.* history-only, healthy *v.* depression
Feeding method at 8 weeks, breast, *n* (%)	30 (61.2)	13 (54.2)	21 (44.7)	*χ*^2^(2) = 3.33, *P* = 0.50	–
Any feeding problems, yes, *n* (%)	11 (42.3)	10 (43.5)	12 (33.3)	*χ*^2^(2) = 0.80, *P* = 0.67	–
Antidepressant usage at 8 weeks, yes, *n* (%)	0 (0)	5 (20.8)	15 (31.9)	***χ*^2^(2) = 17.65, *P*** **< 0.001**	Healthy *v.* history-only, healthy *v.* depression
SCID diagnosis of MDD between birth and 8 weeks, yes, *n* (%)	1 (2.0)	3 (10.7)	11 (21.2)	**χ^2^(2) = 9.37, *P*** **= 0.009**	Healthy *v.* depression
BDI score at 8 weeks, mean (s.d.)	3.02 (3.0)	4.67 (3.6)	11.33 (9.9)	***F*(2, 111) = 19.22, *P*** **< 0.001**	Healthy *v.* depression, history-only *v.* depression
STAI-S score at 8 weeks, mean (s.d.)	25.91 (7.5)	33.96 (9.2)	41.82 (13.6)	***F*(2, 110) = 25.50, *P*** **< 0.001**	Healthy *v.* history-only, healthy *v*.depression,, history-only *v.* depression
Infant age in days at 12-month assessment, mean (s.d.)	396.90 (22.1)	421.80 (42.0)	402.80 (27.6)	***H*_(2)_ = 7.97, *P* =** **0.019**	Healthy *v.* history-only, history-only *v.* depression
Total months of breastfeeding at 12 months, mean (s.d.)	8.22 (3.9)	6.81 (3.9)	8.01 (3.7)	*F*(2, 89) = 23.45, *P* = 0.45	–
Antidepressant usage at 12 months, yes, *n* (%)	0 (0)	5 (19.2)	16 (34.0)	***χ*^2^(2) = 18.26, *P*** **< 0.001**	Healthy *v.* history-only, healthy *v.* depression
SCID diagnosis of MDD between 8 weeks and 12 months, yes, *n* (%)	2 (4.3)	8 (32.0)	17 (37.8)	***χ*^2^(2) = 15.59, *P*** **< 0.001**	Healthy *v.* history-only, healthy *v.* depression
Any SCID diagnosis of MDD between birth and 12 months, yes, *n* (%)	3 (6.5)	9 (36.0)	20 (44.4)	***χ*^2^(2) = 17.51, *P*** **< 0.001**	Healthy *v.* history-only, healthy *v.* depression
BDI score at 12 months, mean (s.d.)	3.40 (3.3)	6.17 (5.5)	10.95 (11.1)	*F*(2, 100) = 10.04, ***P*** **< 0.001**	Healthy *v.* depression , history-only *v*. depression
STAI-S score at 12 months, mean (s.d.)	27.74 (8.2)	34.10 (9.8)	38.99 (12.1)	*F*(2, 100) = 12.29, ***P*** **< 0.001**	Healthy *v.* history-only, healthy *v.* depression

Bold values indicate statistical significance. SES, socioeconomic score; MDD, major depressive disorder; BDI, Beck Depression Inventory; STAI-S, State-Trait Anxiety Inventory-State; NBAS, Neonatal Behavioural Assessment Scale; SCID, Structured Clinical Interview for DSM-IV Disorders.

### Mother–infant interaction assessment

The dyadic relationship was assessed with the Crittenden Child-Adult Relationship Experimental Index (CARE-Index),^29^ a reliable and valid coding method used both clinically and in research with infants up to 15 months of age. We filmed 3-min interactions at 8 weeks and 12 months postnatal, for which mothers were instructed to play with their babies as they normally would. Quality of the interaction was rated across three scales: maternal behaviour, infant behaviour and dyadic synchrony. Mothers and infants were given scores based on seven aspects of behaviour: facial expression, vocal expression, position and body contact, affection and arousal, turn-taking contingencies, control and choice of activity.

The CARE-Index classes maternal behaviour into the following patterns: sensitivity, control and unresponsiveness. Sensitivity is defined as any pattern of behaviour that ‘pleases the infant and increases the infant's comfort and attentiveness and reduces its distress and disengagement’;^30^ the two inverse behaviours of sensitivity are control, indicating intrusiveness or hostility, and unresponsiveness, indicating withdrawal from/unconnectedness to the baby. Likewise, infant behaviour is classed into cooperativeness (being pleasantly connected to the mother) and three inverse behaviours: compulsiveness, presenting as fear or compliance; difficultness, presenting as negative connectedness and over-arousal; and passiveness, presenting as withdrawn and affectless behaviour. Both members can express behaviours across all patterns, but will typically display a dominant pattern. Patterns were scored from 0 to 14, where a higher score reflected greater presence of that behaviour.

Finally, dyadic synchrony is a global indication of how in-tune the dyad is, and how well they are interacting; that is, the overall quality of the interaction. It is rated 0–14, and divided into four categories, with suggested corresponding interventions when used clinically: risk (0–4, ‘need for parental psychotherapy and possible parent–child separation’); inept (5–6, ‘voluntary parent–infant intervention’); adequate (7–10, ‘optional parent education, but no intervention necessary’) and sensitive (11–14, ‘no intervention necessary’). As dyadic synchrony is highly correlated with maternal sensitivity and infant cooperativeness, the present study only reports on dyadic synchrony. Additionally, as behavioural patterns are scored proportionally to each other, the present study only includes mothers’ unresponsiveness (negatively correlated with control) and infants’ compulsivity and passiveness (negatively correlated with difficultness).

The primary trained rater (R.H.B.) has achieved level II reliability with the CARE-Index course (mean correlation coefficient of 0.85 across all scales); reliability was also established between trained raters (blind to maternal mental health status), with an interclass correlation coefficient of 0.90 for dyadic synchrony at 8 weeks and 0.95 at 12 months.

### Statistical analysis

Analyses were conducted with SPSS Statistics version 24 for MacOS (IBM, UK). Before analyses, data were checked for missing data, outliers, accuracy and normality. Main assumptions of normality and homoscedasticity were tested to ensure representativeness of our sample. If violated, data were either log-transformed or non-parametric analyses were conducted. Pearson's *χ*^2^ was used for categorical data, including sociodemographic and clinical variables, and the *z*-test was used to compare the three groups. ANOVA was used to compare means for sociodemographic and clinical variables. Kruskal–Wallis test was used to compare means for the mother–infant interaction, with Bonferroni corrections for *post hoc* comparisons between each of the groups. Analysis of covariance was used for covariate analyses of the interaction. Finally, hierarchical linear regressions were used for prediction modelling for dyadic synchrony. Means (s.e.) are presented in graphs.

## Results

### Sample characteristics

#### Antenatal risk factors

Antenatal characteristics are presented in Table 1. In terms of antenatal clinical symptoms, women in the depression group, by definition, reported higher symptoms of depression and anxiety on the BDI and STAI (*P* < 0.001 for all comparisons); moreover, women in the history-only group displayed more anxious symptoms on the STAI than women in the healthy group (*P* = 0.013). Furthermore, similar proportions in both the depression and history-only groups took antidepressants in pregnancy (37% and 21%, respectively) before or after the baseline assessment. With regard to past depression, women in the depression and history-only groups were equally likely to have a history of recurrent depression before pregnancy, defined as two or more prior episodes (58% and 63%, respectively).

Table 1 also presents the aforementioned SES and clinical risk factors for antenatal and/or postnatal depression in the three groups.^6^ Women in both the depression and history-only groups had similar rates of exposure to childhood maltreatment, both of which were higher than women in the healthy group (63% and 52% *v.* 16%, respectively; *P* < 0.001). However, only women in the depression group were also more likely to have lower education qualifications (versus women in the healthy group), be single (versus women in the history-only and healthy groups), be unemployed (versus women in the healthy group), belong to an ethnic minority group (versus women in the history-only group) and, above all, have a lower composite SES score (versus women in the healthy group); that is, a score encompassing age, ethnicity, marital status, occupation and qualification level at baseline (*P* = 0.001–0.04; see Table 1). Because of the group differences in history of childhood maltreatment and SES score, they were included in subsequent univariate correlations and hierarchical regression models predicting dyadic synchrony (see Tables 2 and 3).

**Table 2:** Correlations between maternal and infant variables with group differences and dyadic synchrony

	Dyadic synchrony at 8 weeks	Dyadic synchrony at 12 months
SES score^a^	0.29**	0.38**
History of maternal childhood maltreatment^b^	−0.11	−0.14
BDI score during pregnancy^a^	0.01	−0.06
STAI-S score during pregnancy^a^	−0.06	−0.16
Antidepressants during pregnancy^b^	−0.17	−0.17
NBAS orientation score^a^	0.20*	0.25**
Postnatal MDD birth to 8 weeks^b^	−0.19*	0.01
BDI score at 8 weeks^a^	0.06	−0.02
STAI-S score at 8 weeks^a^	−0.07	−0.23*
Antidepressants at 8 weeks^b^	−0.06	−0.09
Postnatal MDD at 8 weeks^b^	0.01	−0.10
BDI score at 12 months^a^	0.01	0.08
STAI-S score at 12 months^a^	0.02	−0.18
Antidepressants at 12 months^b^	−0.07	−0.03
Postnatal MDD 8 weeks to 12 months^b^	0.12	0.03

Bold values indicate statistical significance. SES, socioeconomic score; BDI, Beck Depression Inventory; STAI-S, State-Trait Anxiety Inventory-State; NBAS, Neonatal Behavioural Assessment Scale; MDD, major depressive disorder.

a. Spearman's correlation coefficients are presented.

b. Point-biserial correlation coefficients are presented.

*P < 0.05, **P < 0.01.

**Table 3:** Hierarchical regression for variables predicting dyadic synchrony at 8 weeks and 12 months postnatal

Factors	*R*^2^	Statistical significance of change in *R*^2^	Coefficient (95% CI)
**Dyadic synchrony at 8 weeks (*n* = 109)**			
SES score	0.08	0.003	0.07 (0.03 to 0.12)
SES score plus orientation score	0.13	0.020	0.06 (0.01 to 0.10), 0.04 (0.01 to 0.07)
SES score plus orientation score plus PND	0.13	0.748	0.06 (0.01 to 0.10), 0.04 (0.01 to 0.07), 0.02 (−0.08 to 0.11)
SES score plus orientation score plus PND plus maternal group:	0.16	0.256	0.05 (0.00 to 0.10), 0.03 (−0.01 to 0.06), 0.04 (−0.06 to 0.14)
Healthy group			Reference
Depression group			−0.07 (−0.19 to 0.04)
History-only group			−0.10 (−0.22 to 0.03)
**Dyadic synchrony at 12 months (*n* = 103)**			
SES score	0.20	<0.001	0.10 (0.06 to 0.14)
SES score plus orientation score	0.20	0.406	0.09 (0.05 to 0.13), 0.01 (−0.02 to 0.04)
SES score plus orientation score plus PND	0.20	0.834	0.09 (0.05 to 0.13), 0.01 (−0.02 to 0.04), 0.01 (−0.07 to 0.09)
SES score plus orientation score plus PND plus maternal group:	0.23	0.248	0.09 (0.05 to 0.13), 0.00 (−0.03 to 0.03), 0.03 (−0.06 to 0.12)

Healthy group			Reference
Depression group			−0.06 (−0.16 to 0.05)
History-only group			−0.09 (−0.20 to 0.02)

SES, socioeconomic score; PND, postnatal depression.

#### Postnatal outcomes

Postnatal characteristics are also presented in Table 1. There were no statistically significant group differences in mode of delivery, infant sex, feeding method or problems, or total duration of breastfeeding.

We evaluated the prevalence of PND by using the SCID-I across three time points: birth to 8 weeks, 8 weeks to 12 months and birth through 12 months. Overall, women in both the depression and history-only groups were more likely to experience PND in the 12-month postnatal period than women in the healthy group (44% and 36% *v.* 7%, respectively; *P* < 0.002). Between birth and 8 weeks, women in the depression group had the highest rates of PND, followed by women in the history-only and healthy groups (21% *v.* 11% *v.* 2%, respectively; *P* = 0.009), but between 8 weeks and 12 months, women in the depression and history-only groups had similar rates (38% and 32% *v.* 4%, respectively; *P* < 0.01). As this variable differed between the groups, it was included in subsequent univariate correlations and hierarchical regression models predicting dyadic synchrony (see Tables 2 and 3).

Finally, NBAS orientation abilities (the ability for the infant to attend to auditory and visual stimuli at 6 days) was significantly poorer in infants of women in both the depression and history-only groups than in infants of women in the healthy group (6.3 ± 1.5 and 6.4 ± 1.7 *v.* 7.7 ± 0.9, respectively; *F*(2, 127) = 14.6; *P* < 0.001; Tukey *post hoc* test *P* < 0.001 for both comparisons versus healthy group; *P* = 0.94 between depression and history-only groups). As this variable differed between the groups, it was included in subsequent univariate correlations and hierarchical regression models predicting dyadic synchrony (see Tables 2 and 3).

### Dyads in the depression and history-only groups have less optimal mother–infant interaction at both 8 weeks and 12 months postnatal

**Fig. 2:**
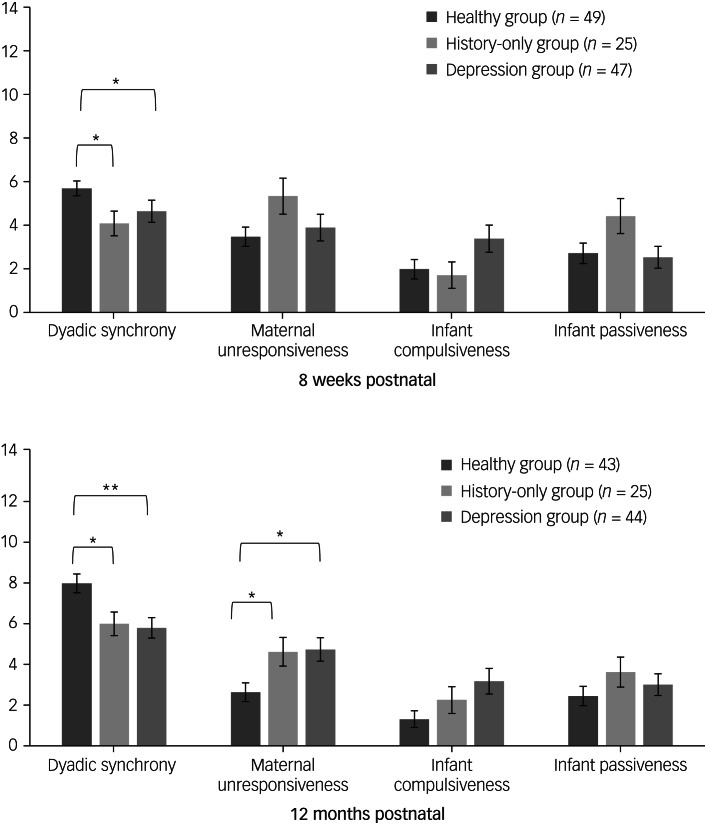
Mean scores across patterns of behaviour on the Crittenden Child-Adult Relationship Experimental Index at 8 weeks and 12 months postnatal. *P < 0.05, **P < 0.01.

We compared scores on the CARE-Index between dyads in the depression, history-only, and healthy groups, at 8 weeks and 12 months postnatal (see Fig. 2).

Dyads in both the depression and history-only groups had lower dyadic synchrony scores compared with the healthy group, at both 8 weeks (4.6 ± 3.5 and 4.3 ± 2.9 *v.* 5.7 ± 2.4, respectively) and 12 months (5.6 ± 3.2 and 5.8 ± 2.8 *v.* 7.7 ± 2.9, respectively), with no difference between the depression and history-only groups (*H*_(2)_ = 9.06, *P* = 0.011 at 8 weeks and *H*_(2)_ = 12.85, *P* = 0.002 at 12 months; Bonferroni correction *P* = 0.026 (healthy *v*. depression), *P* = 0.048 (healthy *v*. history-only) and *P* = 0.002 (healthy *v*. depression), *P* = 0.038 (healthy *v*. history-only), respectively). Notably, at 8 weeks, 62% of dyads in the depression group and 56% of dyads in the history-only group scored in the risk category (dyadic synchrony of 0–4, clinically indicating a need for mother–infant therapeutic interventions^30^), compared with 37% of dyads in the healthy group (*χ*^2^(2) = 6.38, *P* = 0.041); and at 12 months, 48% of dyads in the depression group and 32% of dyads in the history-only group continued to score in the risk range, compared with 14% of dyads in the healthy group (*χ*^2^(2) = 11.56, *P* = 0.003).

In addition, at 12 months, both women in the depression and history-only groups were significantly more unresponsive (higher scores) than women in the healthy group (4.6 ± 3.7 and 4.4 ± 3.4 *v.* 2.5 ± 2.9, respectively; *H*_(2)_ = 9.30; *P* = 0.010; Bonferroni correction *P* = 0.020 (healthy *v*. depression) and 0.045 (healthy *v*. history-only), respectively), again with no difference between women in the depression and history-only groups. There were no other differences in patterns of behaviour between groups.

Of note, infants of the depression and history-only groups were significantly older than those of the healthy group at both the 8-week and 12-month assessments (see Table 1), because of difficulties in organising visits for these mothers because of their increased vulnerability and complexity; however, we repeated the 8-week and 12-month analyses on dyadic synchrony with infant age as a covariate, and our results did not change (*F*(2, 114) = 4.47, *P* = 0.014 at 8 weeks; *F*(2, 105) = 2.43, *P* = 0.010 at 12 months, respectively).

### Variables predicting dyadic synchrony at 8 weeks and 12 months

To determine whether clinical and sociodemographic variables contributed to the disrupted dyadic synchrony in conjunction with depression, we first identified variables that differed between the three groups (see Table 1) and are hypothesised to have a role in mother–infant interaction, according to previous literature. We then conducted univariate correlations between these variables and dyadic synchrony. As such, variables that were correlated with dyadic synchrony included maternal SES score, infant NBAS orientation score at 6 days, maternal STAI score at 8 weeks and maternal presence of PND between birth and 8 weeks (see Table 2). Contrary to hypotheses, and surprisingly, maternal history of childhood maltreatment was not associated with dyadic synchrony, and was thus excluded from further analyses. Furthermore, as maternal STAI score and the presence of PND were highly correlated with each other (*r* = 0.41, *P* < 0.001), we chose to only include PND in follow-up analyses because of its presence in the literature.

Thus, the final variables included in a hierarchical regression predicting dyadic synchrony at 8 weeks and 12 months were as follows: maternal SES score (encompassing age, ethnicity, qualification, employment and marital status), infant NBAS orientation score and presence of PND between birth and 8 weeks. The hierarchical regression models for dyadic synchrony at 8 weeks and 12 months are presented in Table 3.

At 8 weeks, SES score was significantly associated with dyadic synchrony and accounted for 8% of the variance; furthermore, NBAS orientation score was also significantly associated with dyadic synchrony and increased the variance explained to 13%. However, the additions of PND and maternal group (depression/history-only/healthy) did not further influence dyadic synchrony, and the total model explained 16% of the variance.

At 12 months, SES score was still significantly associated with dyadic synchrony and accounted for 20% of the variance; however, neither NBAS orientation, PND nor maternal group further influenced dyadic synchrony, and the total model explained 23% of the variance.

## Discussion

This study examines the quality of mother–infant interactions, both early and late in the postnatal period, in healthy women, women with clinically significant depression in pregnancy and women with a lifetime history of depression but healthy pregnancies. We demonstrate that both depression groups have reduced dyadic synchrony at both 8 weeks and 12 months postnatal, as well as increased maternal unresponsiveness at 12 months postnatal. Contrary to previous literature, in univariate corelations, childhood maltreatment does not affect the mother–infant interaction in our sample, and PND may only have a marginal effect if occurring in the first 8 weeks postnatal. Of the clinical and sociodemographic factors in our sample that could explain these findings, maternal socioeconomic difficulties and dysregulation of neonatal orientation skills (ability to attend to auditory and visual stimuli, indicative of readiness for social interaction) appear to be the most likely contributory factors to reduced dyadic synchrony.

### Antenatal depression and mother–infant interaction

Our findings in women with depression in pregnancy extend and confirm the previous evidence. Although no studies investigating antenatal depression have assessed dyadic synchrony, studies have found increased maternal unresponsiveness, even if measured just at a single assessment, late in the postnatal period;^9,10^ moreover, like ours, these studies measured both antenatal and postnatal depression, and found that the effects of antenatal symptoms are independent of, or stronger than, postnatal symptoms. As our study assesses dyadic synchrony in addition to maternal and infant domains of behaviour, both early and late in the postnatal period, our findings are both consistent with previous literature and novel. Interestingly, we also find that mothers in the depression group are more unresponsive at 12 months, but not at 8 weeks. This suggests that early on, women who are less sensitive are a mix between intrusive/controlling and withdrawn/unresponsive, but over time they become more withdrawn/unresponsive, perhaps because of prolonged depression or a new onset of PND after the first 8 weeks.

### Lifetime history of depression and mother–infant interaction

Compared with healthy women, women with a lifetime history of depression but healthy pregnancies also have disrupted mother–infant interactions, with scores on the CARE-Index overlapping those of women in the depression group. To our knowledge, only one other study has explored this, which found that indeed parents (mothers and fathers) with histories of depression are more likely to show negative affect with their infants and less likely to show positive affect; again, this effect was independent of any postnatal depressive symptoms.^11^ The authors postulated that these findings might be a result of some of these mothers having had depression in pregnancy or early in the postpartum, which is something that they did not assess; however, our results show that the effects of a history of depression are present in the absence of either of these risk factors, as women in the history-only group in our study display minimal depressive symptoms at those time points. One possible neurobiological explanation for these persistent effects of past depression is changes in the levels of circulating oxytocin, a vital hormone for the formation of the mother–infant bond,^12^ as studies have found that women with depression (outside the perinatal period) have dysregulated oxytocin.^13^

### Variables predicting dyadic synchrony

Our hierarchical regression models show that, at both 8 weeks and 12 months, the effect of group (depression/history-only/healthy) on dyadic synchrony is no longer significant upon consideration of the effects of maternal SES score and infant NBAS orientation score (at 8 weeks), or maternal SES score alone at 12 months. Indeed other studies have found that maternal sensitivity (a contributor to dyadic synchrony) can be predicted by socioeconomic factors,^17^ and that women of lower socioeconomic statuses may be less likely to partake in distal (visual and auditory) interactions.^31^ Also of note is that previous studies have found similar negative associations between NBAS scores and mother–infant interaction,^19^ such that reduced neonatal orientation is associated with decreased dyadic synchrony, but never in the context of maternal depression. Given that the orientation cluster of the NBAS assesses infants’ abilities to engage in auditory and visual stimuli, such as interacting with the examiner by tracking their face or voice, this finding could indicate a route through which depression-related genetic vulnerability may translate to difficult infant temperament and thus a disrupted interaction, as suggested by studies showing that transmission of maternal depression into offspring behavioural alterations may be genetically driven.^32^ Moreover, it is also important to consider a transactional model of development, whereby the behaviour of each member of the dyad influences the response in the other,^33^ and thus parenting difficulties in mothers and temperament difficulties in infants may perpetuate each other.

Although it is impossible to make any inference on the factors causally responsible for the reduced quality of mother–infant interaction, our findings do suggest that maternal SES status and neonatal behaviour are part of a pathway that associates depression in mothers (both lifetime and specifically in pregnancy) with less-synchronous mother–infant interaction. It is also important to highlight that women in the history-only group had SES scores that were significantly better than those of women in the depression group, whereas dyadic synchrony in both groups is equally disrupted. In fact, the higher SES score of women in the history-only group could represent the presence of protective factors that may have shielded them from becoming depressed during pregnancy (e.g. because they are more likely to be married/cohabiting, have financial security and/or have social support), but may not have been enough to preserve the quality of the interaction. This indicates that SES difficulties alone cannot causally explain our findings. Above all, when examining the effects that maternal psychopathology may have on offspring outcomes, it is important to consider that many women present with multiple vulnerabilities, and thus many factors, such as depression, socioeconomic vulnerability and infant behaviour, often overlap with each other, adding to the complexity of disentangling the effect that specific variables may have.

Our study also found that women in both the depression and history-only groups are more likely to be postnatally depressed than women in the healthy group, consistent with the notion that antenatal depression and a history of depression are both risk factors for PND.^6^ However, presence of PND itself does not contribute to the prediction of dyadic synchrony in the models, or in the univariate correlations when examined from birth through to 12 months, suggesting that women who experience PND alone may be protected in their interactions. Although many studies have looked at women who experience postnatal depression and have found less optimal interactions with their infants via reduced sensitivity and increased unresponsiveness/withdrawal,^5^ the majority of these studies have not taken into account antenatal symptoms and/or a history of depression. The present study, and two others that looked at both antenatal and postnatal symptoms in the context of mother–infant interaction, found that antenatal depression is more predictive than PND in how the dyad will behave,^9,10^ possibly because antenatal depression may lead to disrupted foetal attachment and bonding early in the postpartum. In contrast, the presence of PND in itself does not detract from the mother–infant relationship if optimal bonding is present early on in the postnatal period,^34^ implying that the encouragement and support for the early relationship can buffer against maternal postnatal psychopathology. To date, other studies that have investigated the impact of perinatal depression on offspring outcomes have also found that antenatal depression is more predictive of outcomes, including offspring behavioural problems.^35^ Taken together, it is plausible that the differences found in studies only investigating postnatal symptoms may be at least partly due to antenatal or lifetime symptoms.

Furthermore, with regard to maternal anxiety, we found that antenatal anxiety was not associated with dyadic synchrony at either time point, suggesting that anxiety or stress in pregnancy do not hinder the development of the mother–infant interaction, consistent with previous literature.^36^ Anxiety at 8 weeks postnatal was correlated with dyadic synchrony at 12 months; however, given how closely anxiety was linked to PND, we expect that postnatal anxiety would not have contributed to dyadic synchrony, in the same way that PND did not in our hierarchical regression models.

Another surprising finding in our study is that although both women in the depression and history-only groups had significantly higher rates of childhood maltreatment, this was not associated with dyadic synchrony. Previous literature on maternal childhood maltreatment and mother–infant interaction has been mixed: some studies have found associations with disrupted mother–infant interactions,^16^ and others have not.^37^ Although a history of childhood maltreatment does increase the risk of perinatal depression,^38^ our study suggests that maternal history of childhood maltreatment may only affect subsequent mother–infant interactions because it is a risk factor for psychopathology, and in the absence of psychopathology (arguably, in resilient mothers), it does not in itself have an effect.

### Limitations

Our main limitation is that we may not have captured all of the important psychological factors relevant to the pathway from maternal depression to reduced quality of the mother–infant interaction, especially considering that the best of our regression models only explains 23% of the variance in dyadic synchrony. For example, much of the literature suggests that mothers are likely to mirror their own upbringing,^39^ but we have not evaluated mothers' attachment to their own mothers. Additionally, many studies find a link between foetal attachment status in pregnancy and mother–infant bonding quality in the postnatal period,^7^ but we did not measure foetal attachment. Another limitation is that we had some attrition throughout the study, although it was <10% at each subsequent time point, and we found no differences between mothers who did and did not drop out. Finally, it is important to acknowledge that the sample size in our study was relatively small, and moreover, as the recruitment of the history-only group began at a later stage in the study, there were fewer women in this group than in the healthy and depression groups; although we believe that the difference in dyadic synchrony between the history-only group and the healthy group is still a robust finding given the magnitude of the effect, we recommend replication with a larger sample size.

### Clinical implications and conclusions

The main clinical implication of our findings is that support for dyads at risk of interaction difficulties should begin antenatally, and also be targeted to well mothers with a history of depression, considering that history of depression is a major risk factor for becoming unwell in the perinatal period. At 8 weeks, already many of the dyads in the depression and history-only groups fall into the risk range on the CARE-Index, which, when used clinically, implies the need for parental treatment psychotherapy;^30^ and, furthermore, a significant proportion of these dyads continued to remain at risk at 12 months. Additionally, and perhaps surprisingly, even some healthy dyads, with neither past nor present psychopathology, fall into the inept range of the CARE-Index at 8 weeks postnatal, suggesting it may even be beneficial for all expectant mothers to receive support before giving birth. Moreover, given that the CARE-Index is used clinically within mother and baby units to assess dyads pre- and post-treatment,^40^ as well as in court cases to evaluate infant safety and risk,^41^ our study also contributes to the findings that it is a valid and robust clinical tool that can evaluate the quality and progression of the mother–infant relationship.

As pregnancy is a time when women are in contact with healthcare professionals, it is therefore an important goal to provide support in bonding with the foetus and future infant for all expectant mothers, not just for those who are unwell. This could be achieved by providing mothers with literature on examples of sensitive mothering behaviours, ways to engage with the foetus and with infants, behaviours to expect from an infant and developmental milestones. Additionally, educating mothers on sensitive behaviour, both with the foetus and the infant, could be incorporated into birthing classes and health visits. Finally, our findings highlight the importance of support from perinatal services for identified vulnerable women, especially across the postnatal period, and suggest that interventions proven to help the mother–infant interaction, such as video feedback^42^ and structured mother-baby activities, e.g., art and singing groups,^43,^^44^ should be more widely available. This way, we may be able to break the intergenerational transmission of psychopathology that begins with maternal depression (lifetime or in pregnancy) and may lead to increased mental health problems in offspring via, at least in part, a disrupted mother–infant interaction.

## Data Availability

The data that support the findings of this study are available from the corresponding author, R.H.B., upon reasonable request.

## Supplementary material

For supplementary material accompanying this paper visit http://dx.doi.org/10.1192/bjo.2021.52. click here to view supplementary material
