# Supplementary material for: Mother–infant interaction in women with depression in pregnancy and in women with a history of depression: the Psychiatry Research and Motherhood – Depression (PRAM-D) study
Source: BJPsych Open. 2021 May 25;7(3):e100. doi: 10.1192/bjo.2021.52 (PMC8167851; doi:10.1192/bjo.2021.52)
Supplement: Supplementary file 1 [file bjosup.zip › S2056472421000521sup013.pdf]

**Supplementary table 2**. Sociodemographic and clinical variables at baseline between women who remained in and women who left the study at 8 weeks and 12 months

| | Participated at 8 weeks (n = 121) | Did not participate at 8 weeks (n = 10) | Statistical test | Participated at 12 months (n = 112) | Did not participate at 12 months (n = 19) | Statistical test |
|---|---|---|---|---|---|---|
| SES[a] factor score, mean (SD) | 0.121 | -0.256 | $U$ = 464.0, z = -1.222, p = 0.222 | 0.129 | -0.121 | $U$ = 875.0, z = -1.235, p = 0.217 |
| Parity, primiparous, n (%) | 63 (52.1) | 3 (30.0) | $\chi^2(2) = 2.343$, p = 0.310 | 60 (53.6) | 6 (31.6) | $\chi^2(2) = 4.981$, p = 0.083 |
| Antidepressant usage in pregnancy, yes, n (%) | 21 (17.4) | 4 (40.0) | $\chi^2(2) = 3.067$, p = 0.080 | 22 (19.6) | 3 (15.8) | $\chi^2(2) = 0.156$ p = 0.693 |
| BDI[b] score at baseline, mean (SD) | 9.57 (10.51) | 15.72 (17.11) | t(121) = 1.173, p = 0.266 | 9.83 (11.46) | 11.92 (10.38) | t(121) = 0.707, p = 0.481 |
| STAI-S[c] score at baseline, mean (SD) | 36.92 (14.43) | 43.91 (16.97) | t(121) = 1.510 p = 0.134 | 37.09 (14.70) | 40.37 (15.09) | t(121) = 0.853, p = 0.396 |
| Number episodes of MDD prior to pregnancy, >2, n (%) | 40 (33.3) | 5 (55.6) | $\chi^2(2) = 2.318$, p = 0.314 | 40 (31.0) | 5 (26.3) | $\chi^2(2) = 0.917$, p = 0.632 |
| Maternal history of childhood maltreatment, yes, n (%) | 46 (41.8) | 5 (50) | $\chi^2(2) = 0.251$, p = 0.616 | 44 (41.9) | 7 (46.7) | $\chi^2(2) = 0.122$, p = 0.727 |

[a]Socioeconomic Score
[b]Beck Depression Inventory
[c]State-Trait Anxiety Inventory- State
