# Supplementary material for: Mother–infant interaction in women with depression in pregnancy and in women with a history of depression: the Psychiatry Research and Motherhood – Depression (PRAM-D) study
Source: BJPsych Open. 2021 May 25;7(3):e100. doi: 10.1192/bjo.2021.52 (PMC8167851; doi:10.1192/bjo.2021.52)
Supplement: Supplementary file 1 [file bjosup.zip › S2056472421000521sup014.pdf]

Supplementary table 1. Study attrition by group at 8 weeks and 12 months postnatal

	Healthy (n = 51)	History-only (n = 28)	Depressed (n = 52)	Statistical test and significance
Did not participate at 8 weeks, n (%)	2 (3.9)	3 (10.7)	5 (9.6)	$\chi^2(2) = 2.691$, $p = 0.260$
Did not participate at 12 months, n (%)	8 (15.7)	3 (10.7)	8 (15.8)	$\chi^2(2) = 0.414$, $p = 0.813$
